# Applications of ambient ionization mass spectrometry in 2022: An annual review

**DOI:** 10.1002/ansa.202300004

**Published:** 2023-04-26

**Authors:** Stephanie Rankin‐Turner, Patrick Sears, Liam M Heaney

**Affiliations:** ^1^ W. Harry Feinstone Department of Molecular Microbiology and Immunology, Johns Hopkins Bloomberg School of Public Health Johns Hopkins University Baltimore Maryland USA; ^2^ School of Chemistry and Chemical Engineering University of Surrey Guildford UK; ^3^ School of Sport, Exercise and Health Sciences Loughborough University Loughborough UK

## Abstract

The development of ambient ionization mass spectrometry (AIMS) has transformed analytical science, providing the means of performing rapid analysis of samples in their native state, both in and out of the laboratory. The capacity to eliminate sample preparation and pre‐MS separation techniques, leading to true real‐time analysis, has led to AIMS naturally gaining a broad interest across the scientific community. Since the introduction of the first AIMS techniques in the mid‐2000s, the field has exploded with dozens of novel ion sources, an array of intriguing applications, and an evident growing interest across diverse areas of study. As the field continues to surge forward each year, ambient ionization techniques are increasingly becoming commonplace in laboratories around the world. This annual review provides an overview of AIMS techniques and applications throughout 2022, with a specific focus on some of the major fields of research, including forensic science, disease diagnostics, pharmaceuticals and food sciences. New techniques and methods are introduced, demonstrating the unwavering drive of the analytical community to further advance this exciting field and push the boundaries of what analytical chemistry can achieve.

**List of Abbreviations**
AIMS: ambient ionization mass spectrometry
APADI: atmospheric pressure arc desorption/ionization
APCI: atmospheric pressure chemical ionization
ASAP: atmospheric pressure solids analysis probe
CBS: coated blade spray
cVSSI: vibrating sharpedge spray ionization
DART: direct analysis in real time
DBD: dielectric barrier discharge
DESI: desorption electrospray ionization
EAPSI: electroactive polymer‐based spray ionization
EESI: extractive electrospray ionization
ESI: electrospray ionization
FAPA: flowing atmospheric pressure afterglow
FFO: fully formulated oil
FFPE: formalin‐fixed, paraffin‐embedded
HCD: hollow cathode discharge
HPD: heat pulse desorption
iEESI: internal extractive electrospray ionization
ILSA: inverted library search algorithm
IMS: ion mobility spectrometry
LAESI: laser ablation electrospray ionization
LAMP: laser‐assisted micro‐pyrolysis
LC: liquid chromatography
LESA: liquid extraction surface analysis
LMJ‐SSP: liquid micro junction‐surface sampling probe
LOD: limit of detection
LTP: low‐temperature plasma
MALDI: matrix‐assister laser desorption ionization
MS: mass spectrometry
nano‐DESI: nanospray desorption electrospray ionization
NPSs: new psychoactive substances
PDA: polydopamine
PESI: probe electrospray ionization
PI: post‐photoionization
PIRL: picosecond infrared laser desorption
PLS‐DA: partial least squares‐discriminant analysis
PS: paper spray
REIMS: rapid evaporative ionization mass spectrometry
SESI: secondary electrospray ionization
sfPESI: sheath‐flow probe electrospray ionization
TB‐ITP‐DESI: thread‐based isotachophoresis desorption electrospray ionization
THC: tetrahydrocannabinol
TNT: trinitrotoluene
VOC: volatile organic compound

## 1 INTRODUCTION

Mass spectrometry (MS) has played a critical role in the scientific community for decades, finding utility in clinical diagnostics, forensic science, environmental testing, drug discovery, and more. Despite the importance of MS, traditional approaches have long since been hindered by method limitations, necessitating destructive and time‐consuming sample preparation and sometimes lengthy chromatographic separation, all whilst being confined to the laboratory bench. In the mid‐2000s, the field of MS was set to rapidly transform as the first ambient ionization techniques were introduced. Desorption electrospray ionization (DESI)^1^ and direct analysis in real‐time (DART)^2^ were unveiled somewhat simultaneously, and for the first time enabled the rapid desorption and ionization of native state samples under ambient conditions.

Since then, the field of ambient ionization MS (AIMS) has experienced impressive growth, evident through the dozens of novel AIMS techniques that have been developed, the increasing number of fields realizing and leveraging the power of AIMS, and the constant improvement of methods and technologies. Each year brings the introduction of new techniques aiming to fill a gap in a scientist's analytical toolbox. These advancements in AIMS technologies provide the opportunity to discover novel ways to solve problems, both in the laboratory and, in more recent years, by taking the instrument to the sample in real‐world arenas. AIMS is rapidly finding its place in clinical medicine to speed up diagnostics, forensic science to preserve evidence and solve crimes more quickly, food quality control to ensure the safety of consumer products, and pharmaceutical sciences to study the behaviour of drugs within the body.

This review will delve into the applications and developments of AIMS throughout 2022, continuing from our previous 2020 and 2021 editions.^3^, ^4^ Search terms used include ‘ambient ionization’ and individual AIMS technique names using the PubMed database in October and November 2022. The subsequent sections briefly introduce the reader to key AIMS techniques, followed by a detailed review of AIMS applications in several major fields of research, culminating in a look at recent developments and what the future may hold for AIMS.

## 2 AMBIENT IONIZATION MS

A comprehensive collection of AIMS techniques has been gradually developed over the past 20 years, each utilizing different ion source geometries and ionization mechanisms geared towards the desorption and ionization of different types of molecules. Despite the diversity of AIMS, the majority of techniques broadly fall into three primary classes of technique. Plasma‐based ionization techniques utilize an electrical discharge to produce reactive ions to achieve ionization, similar to traditional atmospheric pressure chemical ionization (APCI) techniques. Solid‐liquid extraction techniques involve the extraction or desorption of analytes directly from the surface of a sample, subsequently achieving ionization using mechanisms akin to electrospray ionization (ESI). Finally, laser‐based ionization techniques involve the ablation and desorption of analytes using infrared or ultraviolet lasers, albeit these techniques are perhaps lesser used than the plasma‐ and surface‐based approaches.

Plasma‐based ion sources are one of the primary classes of AIMS techniques, utilizing electrical discharges to produce a reactive plasma of electrons, radicals and excited state (metastable) species. Within this category, ion sources might be considered indirect in which the electrical discharge itself is physically separated from the sample, or direct where the sample makes direct contact with the excited species.^5^ DART is undoubtedly the most commonly used indirect plasma‐based AIMS technique, developed in the mid‐2000s by Cody et al.^2^ In DART, an inert gas, typically helium or nitrogen, enters the DART ion source where an electrical discharge occurs, resulting in the production of the ionizing plasma. Various ion‐molecule reactions then occur, resulting in the ionization of either atmospheric reagent ions that subsequently ionize the analyte molecules or, in some cases, the immediate ionization of analyte molecules. Due to the commercial availability of DART, its ease of operation, and its broad applicability to gases, liquids and solids, this technique has rapidly become one of the most widely used, particularly for the analysis of materials of forensic relevance. Another commercially‐available device is the atmospheric pressure solids analysis probe (ASAP), which utilizes a glass sampling tool that is directly exposed to the sample in order to collect material for analysis.^6^ The probe is then inserted into the instrument where a stream of heated gas induces the rapid thermal desorption of volatile and semi‐volatile analytes, followed by ionization via APCI. The simplicity of ASAP has also made it a popular technique, with great potential for use by non‐experts, especially given the 2021 launch of the first dedicated AIMS mass spectrometer.

Despite the availability of commercialized plasma‐based AIMS devices, these ion sources can also be readily handmade at a relatively low cost. Dielectric barrier discharge (DBD) ionization consists of two electrodes, for instance, a copper strip and a stainless steel needle, with an insulating barrier (e.g. glass) placed in between.^7^ A high voltage is applied between the electrodes, resulting in the formation of a plasma that can be applied directly to gaseous, liquid or solid samples. Other similar plasma‐based techniques include low‐temperature plasma (LTP), hollow cathode discharge (HCD), plasma‐assisted desorption ionization, and flowing atmospheric pressure afterglow (FAPA), which operate under similar principles but with modified geometries and mechanisms.^8^ Although many plasma‐based ion sources are not commercially available, they do offer the advantage of being relatively cheap and simple to construct. For instance, DBD ion sources simply require a glass or quartz tube, two electrodes, and a high‐voltage power supply, which can subsequently be coupled with any open‐inlet mass spectrometer. Plasma‐based ion sources are amongst the few AIMS techniques that can be readily applied to gaseous, liquid and solid samples alike.

**FIGURE 1:**
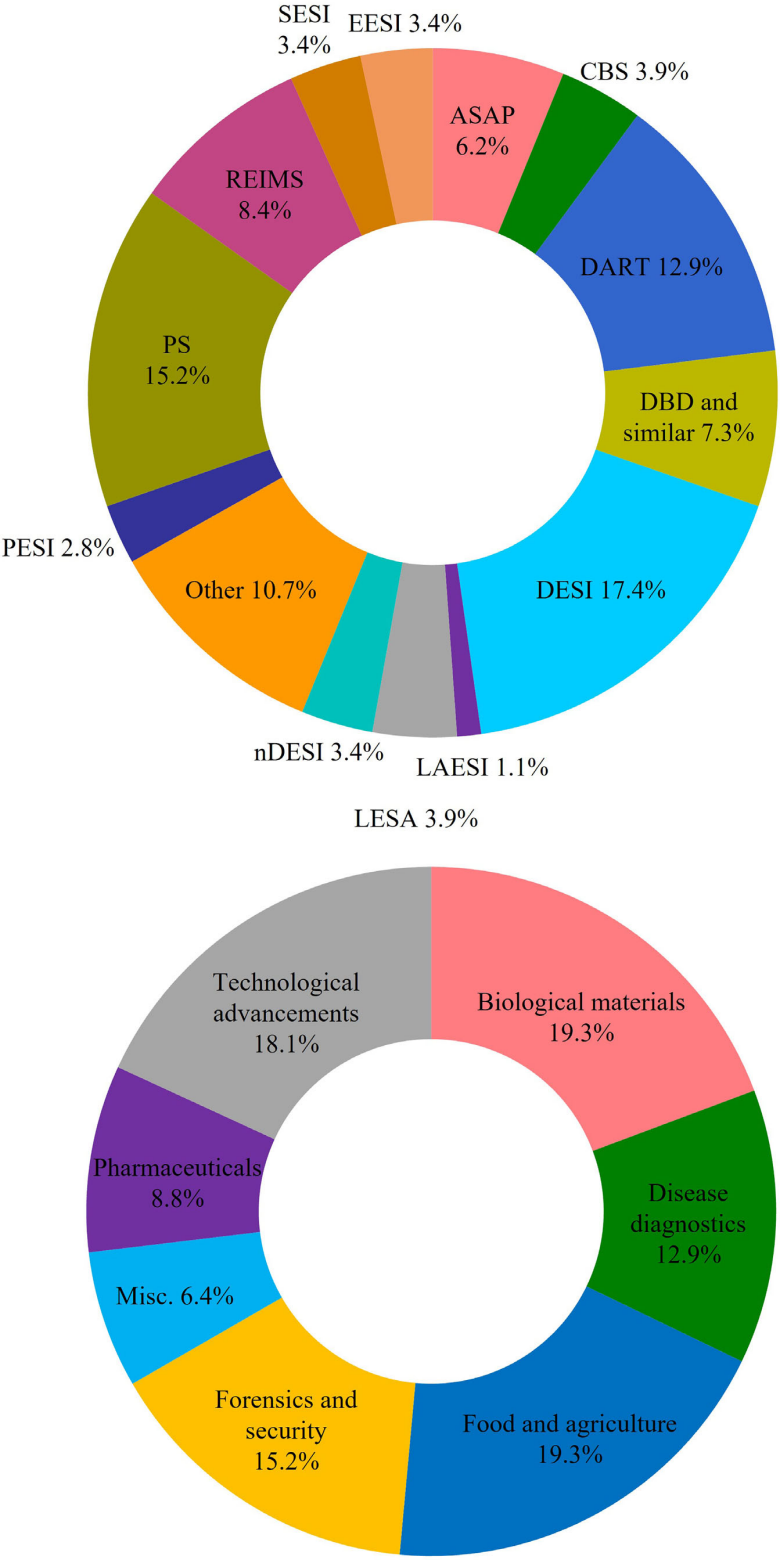
Distribution of papers detailed in this review using each ambient ionization mass spectrometry (AIMS) technique (above) and the relative contribution of papers to each field of research (below). Please see Table 1 for details on the abbreviations used for AIMS techniques.

Solid‐liquid extraction AIMS techniques account for the largest proportion of publications in AIMS covered in this review (Figure 1), with DESI being the first to be introduced. DESI uses an electrically‐charged stream of microdroplets which, upon collision with the surface of a sample, results in the production of secondary droplets containing desorbed and ionized molecules.^1^ The ions are then drawn directly into the nearby MS inlet for detection. Since its advent, numerous modified forms of DESI have emerged aiming to resolve some of the weaknesses of the initial technique and extend its capabilities. These include nanospray DESI (nano‐DESI), which utilizes two small capillaries to create a liquid microjunction with the surface of the sample for localized analyte extraction,^9^ and air flow‐assisted DESI, which incorporates a high‐flow air stream to improve analyte extraction and transportation.^10^ This collection of DESI‐based techniques has proven to be advantageous for the MS imaging of biological materials, particularly given its high spatial resolution.^11^

Paper spray (PS) ionization is also amongst the most commonly utilized AIMS techniques, accounting for 15% of publications covered in this review. This technique consists of a triangular piece of paper substrate that is positioned directly in front of the MS inlet.^12^ A small volume of the sample and a spray solvent is added, followed by the application of a high voltage. This results in electrospray occurring at the pointed tip of the paper, leading to the ionization of analytes with similar mechanisms to traditional ESI. PS‐MS has been rapidly adopted by many research laboratories worldwide due to its low cost, ease of use, and versatility in terms of the paper substrate used for the analysis. Each year novel paper treatments and variations are introduced, expanding the applicability of this technique to different challenges. A three‐dimensional version of PS, known as cone spray, has become a popular technique for the analysis of solids,^13^ and the use of alternative substrates and paper treatments to target different molecule types and induce on‐paper reactions has vastly expanded the possibilities of analysis using PS.^14^ Similar techniques have even been produced directly from the sample material itself, such as leaf spray ionization.^15^ PS‐MS has rapidly become one of the most commonly utilized AIMS techniques, driven by its relatively low cost, versatility, and low detection limits, at times achieving impressive parts per trillion levels of detection.^16^

Probe ESI (PESI) is another popular and now commercialized AIMS technique, first developed in the mid‐2000s shortly after the introduction of DESI and DART.^17^ In PESI, a grounded solid needle is lowered onto the surface of a liquid, collecting a small amount of material on the end of the needle. The needle is then raised in front of the mass spectrometer inlet and a high voltage is applied, initiating the formation of an electrospray from the needle tip. As the original PESI technique was only applicable to liquids and soft solid samples, it was later modified to produce sheath‐flow PESI (sfPESI).^18^ In sfPESI, the needle is housed in a plastic solvent‐filled capillary which protrudes from the base of the capillary to enable the liquid extraction of solid surfaces. This has greatly expanded the application of PESI to materials such as agricultural products^19^ and dried blood spots.^20^

Somewhat comparable to sfPESI is liquid extraction surface analysis (LESA), an AIMS technique that extracts analytes via a liquid microjunction created between the conductive pipette tip of a sampling probe and the sample surface.^21^ Analytes are extracted from the sample into the liquid through repeated re‐aspiration of the liquid against the sample surface. The pipette tip is then repositioned in front of the instrument inlet and electrospray is induced. LESA has grown into a particularly promising technique for the imaging of biological tissues. Somewhat analogous to LESA is the MasSpec Pen, another technique which utilizes liquid‐solid extraction of analytes prior to analysis.^22^ In particular, the MasSpec Pen has found its utility in the real‐time analysis of biological tissues, particularly in operating theatres, though in recent years more diverse applications have emerged. Techniques such as the MasSpec Pen have particular promise for real‐world deployment, requiring minimal expertise to operate. Extractive ESI (EESI) utilizes two colliding aerosols, one containing the nebulized sample solution and the other a spray solvent with a high voltage applied to produce charged microdroplets.^23^ As the streams collide, charge transfer and ionization occur. Finally, although not a solid‐liquid extraction technique, secondary ESI (SESI) is a technique most akin to the aforementioned electrospray‐based techniques.^24^ SESI incorporates a sprayer to produce charging agents which collide with gas‐phase analytes, after which charge transfer and ionization of analytes take place, with ions being drawn directly into the MS inlet for detection.

When considering laser‐based AIMS techniques, perhaps the most common is laser ablation ESI (LAESI).^25^ In LAESI, the sample surface is ablated using an infrared laser, producing a plume of desorbed molecules which are subsequently ionized by electrospray. Laser ablation techniques are particularly suited to MS imaging due to their ability to achieve extremely focused ablation and sampling. A particular advantage of laser ablation is the potential to achieve extremely fast analysis times, with some authors demonstrating the analysis of dozens of samples per second.^26^, ^27^ Finally, some AIMS techniques utilize somewhat unique mechanisms and do not fall within the aforementioned classes. Rapid evaporative ionization MS (REIMS) is one such technique, which uses a surgical electrocautery knife to achieve vaporization of the sample.^28^ The gaseous plume containing analyte ions is then drawn into the mass spectrometer for analysis. REIMS, sometimes referred to as the iKnife, was primarily developed for use in operating theatres by surgeons to guide the removal of cancerous tissue based on the respective chemical profiles of malignant and healthy tissue.

The selection of techniques discussed here, included in Table 1, is by no means exhaustive but encompasses the major AIMS techniques commonly encountered and those utilized in studies covered in this review. The subsequent sections describe the diverse uses of these techniques throughout 2022 across several key fields of research and highlight the development of new methodological and technological developments driving the field forward.

**TABLE 1:** Summary of techniques covered throughout this review

**Technique**	**Abbreviation**	**Classification**
Atmospheric breath analysis probe	ABAP	Plasma
Air flow‐assisted desorption electrospray ionization	AFADESI	Liquid extraction
Air flow‐assisted ionization	AFAI	Liquid extraction
Atmospheric pressure arc desorption/ionization	APADI	Plasma
Atmospheric pressure chemical ionization	APCI	Plasma
Atmospheric pressure solids analysis probe	ASAP	Plasma
Coated blade spray	CBS	Liquid extraction
Cone spray ionization	CSI	Liquid extraction
Vibrating sharpedge spray ionization	cVSSI	Liquid extraction
Desorption atmospheric pressure chemical ionization	DAPCI	Plasma
Direct analysis in real time	DART	Plasma
Dielectric barrier discharge	DBD	Plasma
Desorption electro‐flow focusing ionization	DEFFI	Liquid extraction
Desorption electrospray ionization	DESI	Liquid extraction
Desorption, separation and ionization	DSI	Plasma
Electroactive polymer‐based spray ionization	EAPSI	Liquid extraction
Extractive electrospray ionization	EESI	Liquid extraction
Flowing atmospheric pressure afterglow	FAPA	Plasma
Heat‐assisted dual neutral spray ionization	HADSI	Liquid extraction
Hollow cathode discharge	HCD	Plasma
Handheld liquid microjunction‐surface sampling probe	hLMJ‐SSP	Liquid extraction
Heat pulse desorption	HPD	Plasma
Internal extractive electrospray ionization	iEESI	Liquid extraction
Laser ablation electrospray ionization	LAESI	Laser ablation
Liquid extraction surface analysis	LESA	Liquid extraction
Liquid micro junction‐surface sampling probe	LMJ‐SSP	Liquid extraction
Low‐temperature plasma	LTP	Plasma
MasSpec Pen	–	Liquid extraction
Nanospray desorption electrospray ionization	nano‐DESI	Liquid extraction
Plasma‐assisted desorption ionization	PADI	Plasma
Probe electrospray ionization	PESI	Liquid extraction
Picolitre pressure probe electrospray ionization mass spectrometry	picoPPESI	Liquid extraction
Picosecond infrared laser desorption	PIRL	Laser ablation
Paper spray ionization	PS	Liquid extraction
Rapid evaporative ionization mass spectrometry	REIMS	Other
Secondary electrospray ionization	SESI	Other
Sheath‐flow probe electrospray ionization	sfPESI	Liquid extraction
Thread‐based isotachophoresis desorption electrospray ionization	TB‐ITP‐DESI	Liquid extraction
Thread spray ionization	TSI	Liquid extraction

## 3 APPLICATIONS OF AIMS

### 3.1 Forensics and security

The development of MS techniques suitable for rapid, on‐site analysis has naturally gained considerable interest in the forensic science community. Given the nature of forensic evidence, the ability to quickly characterize materials of potential forensic interest has the capacity to significantly enhance the timeline of criminal investigations. Furthermore, many ambient ion sources have the capability to be coupled with portable mass spectrometers, offering a technology that will be key to improving on‐site analysis at crime scenes.

One of the largest applications of AIMS is in the rapid detection of illicit drugs, both as bulk drug products and in body fluids. DART‐MS is amongst the leading AIMS techniques in forensic drug analysis, largely due to its commercial availability, rapid analysis time and non‐destructive nature. Furthermore, DART has been demonstrated to be capable of detecting low levels of drugs, often achieving low parts per billion limits of detection.^29^ Ventura et al. developed a DART technique for the characterization of tryptamine‐based new psychoactive substances (NPSs).^30^ The group created a library of neutral loss spectra acquired from 50 tryptamine structures under different fragmentation conditions. Upon training and validating a partial least squares‐discriminant analysis (PLS‐DA) model, predictions of unknown compounds could be achieved with an accuracy of 100%. Given the variation in fragmentation, and thus mass spectral profiles, that can result from different ionization conditions, the development of libraries across diverse instrumental conditions is an important step in the implementation of AIMS technologies. To further drive AIMS in police laboratories, there are increased demonstrations of the use of DART for the analysis of seized samples. Researchers at the National Institute of Standards and Technology have been developing DART for forensic analysis for several years. In a recent study, they used the technique for the analysis of 92 drug samples seized by the Maryland State Police, implementing their recently developed DART‐MS Forensic Database and inverted library search algorithm (ILSA).^31^ Using real case samples, they demonstrated their ILSA to be suitable for the analysis of both pure compounds and complex mixtures, which are often encountered by forensic laboratories and highlighted best practice for identifying samples using DART spectral libraries. In a recent study by Dubai Police, 188 e‐cigarette samples seized over a 4‐year period were analyzed for the presence of controlled substances.^32^ In total, 84% of samples were positive for controlled substances, with 98% containing tetrahydrocannabinol (a psychoactive component of marijuana, THC) and the remaining samples containing other illicit drugs, including synthetic cannabinoids and amphetamines. Similarly, the US Food and Drug Administration used DART‐MS in a larger study of almost 3000 e‐cigarette samples as part of an investigation into the role of e‐cigarettes in a recent outbreak of vaping‐related lung injuries.^33^ In this study, samples were dissolved in acetonitrile prior to analysis, demonstrating DART to be a powerful tool in the simultaneous detection of multiple target analytes. DART has also been utilized in the analysis of cannabidiol products which, although now legal in several countries, still require close analytical scrutiny to ensure product safety,^34^ to differentiate isomeric novel psychoactive substances,^35^ and for the analysis of psychoactive plant materials.^36^

Whereas ambient ionization has a clear application in the identification of bulk and trace illicit substances, the ability to rapidly screen biological samples, such as blood and urine, is also of great interest. Kim et al. used sfPESI for the rapid detection of cocaine metabolites in dried blood spots.^37^ Benzoylecgonine, cocaethylene and ecgonine methyl ester were all detected in blood spots aged for 48 h, with detection of metabolites achieved in less than 20 seconds per sample with no sample preparation. The study furthermore built upon previous iterations of the sfPESI probe, this time incorporating a continuous flow solvent system to prevent the need for refilling the probe. PS‐MS has additionally been applied to the detection of illicit substances in biofluids, specifically in urine. In a recent study, an automated plate‐based PS‐MS system coupled with a rapid glucuronide hydrolysis step was developed for the semi‐quantitative screening of 40 common drugs of abuse and metabolites in urine.^38^ Up to 240 samples could be analyzed automatically with an analysis time of approximately 2 min per sample, demonstrating the potential of techniques such as PS‐MS to reduce the backlog of biological samples in drug screening labs. Similarly, Borden et al. used a reactive PS‐MS method for the detection of cannabinoids in urine and saliva.^39^ An on‐paper derivatization method was used to increase the sensitivity of the technique, resulting in a limit of detection (LOD) of 0.8 ng/m for THC in saliva and 1.3 ng/ml for carboxy‐THC in urine, the primary metabolite of THC. Finally, Vejar‐Vivar et al. used a hypodermic needle with a polydopamine (PDA)‐coated inner wall as a microextraction and electrospray device to detect methamphetamine, methadone and cocaine in saliva.^40^ The PDA film inside the needle allows for the rapid extraction of analytes at an alkaline pH, then eluted in a methanol‐based ESI solution for ionization. The study presents a cheap and disposable extraction/ionization probe with an overall analysis time of 3 min per sample. Though the detection of illicit substances in body fluids is more typical, Kong et al. presented an AIMS technique for the rapid analysis of drugs in hair samples.^41^ In this technique, a single hair is placed on a metal ceramic heater and a high voltage and solvent are added to the sample. This results in the thermal desorption and ionization of drugs within the hair without the need for pulverization, extraction and chromatographic analysis typically used for hair‐based drug testing. Numerous other AIMS techniques have proven capable in the analysis of controlled substances, including coated blade spray (CBS),^42^ PS‐MS,^43^ and thermal desorption‐assisted DBD.^44^ There is recent evidence of interest in the use of AIMS in anti‐doping, with ASAP being utilized for the analysis of anabolic steroids^45^ and both DESI and PS‐MS for the rapid detection of stimulants and diuretics in urine.^46^

The development of ambient ionization techniques for the analysis of explosives also constitutes a notable proportion of applications in forensic science. The rapid detection and identification of explosives are of crucial importance to national security, and the development of techniques that could feasibly be installed in airports and used on‐site at incident scenes are of particular interest. Li et al. developed a custom‐built simple‐to‐use thermal desorption sampler with a DBD ionization source coupled with a homemade miniature mass spectrometer for explosives analysis.^47^ The technique was applied to the characterization of several common explosives including trinitrotoluene (TNT), octogen, hexogen, and pentaerythritol tetranitrate, achieving detection limits as low as 0.01 ng. Plasma‐based ionization techniques have been particularly popular in the analysis of explosives due to their solvent‐free nature and simple construction.

Gao et al. developed an atmospheric pressure arc desorption/ionization MS (APADI‐MS) technique for the analysis of 10 types of explosives.^48^ In APADI‐MS, the sample is simply placed on a copper substrate over which a needle is positioned. These components make up the arc generator, with an arc plasma forming between the two electrodes. As the explosive samples are exposed to the arc discharge, instant vaporization and ionization occur. The explosives exhibit a high affinity for the nitrate ions produced by the plasma, resulting in the formation of characteristic adduct anions. Hong et al. also utilized a plasma‐based ion source for explosives detection. They developed a variable pressure HCD‐MS method to study gas phase ion‐molecule reactions of multiple nitroaromatic explosives, including TNT, trinitrobenzene, and several dinitrotoluenes.^49^ The technique was validated using air as the gas, taking into consideration the need to use cheap and readily available resources when implementing such techniques in the field. Furthermore, the study specifically focused on the detection of trace levels of explosives, with LODs in the range of 1‐50 pg. Similarly, AIMS has also been used for the detection of smokeless powders, a class of low explosives typically used as a propellant in firearms but also utilized in the preparation of improvised explosive devices. DART‐MS was used in a pilot study of three nitrocellulose‐based smokeless powders.^50^ The technique proved suitable for the detection of glucose trinitrate and cellulose hexanitrate, the monomeric and dimeric subunits of nitrocellulose, enabling the detection of both bulk and consumed smokeless powders.

Although AIMS techniques are primarily applied to drugs and explosives analysis in forensic research, the benefits of rapid MS analysis techniques have been recognized elsewhere. The need for non‐ or minimally‐destructive analysis techniques has become increasingly important in forensic document analysis, and Sun et al. have developed the use of DESI‐MS for this purpose. The use of fingerprints as personal signatures is a common custom in some countries, and as such could be open to fraud. DESI‐MS was used to characterize components in genuine and falsified stamped fingerprints, readily distinguishing the two based on the presence of sweat‐derived compounds.^51^ The same group also applied this technique to the study of artificially aged inks, demonstrating potential time‐dependent changes in the ink and paper that could be beneficial in confirming the age of questioned documents.^52^

DART‐MS has proven to be an advantageous technique across numerous areas of forensic research. Millbern et al. characterized 31 different disperse dyes in fabrics and single threads, comparing DART analysis with a liquid chromatography (LC)‐MS method to demonstrate DART as a suitable technique for dye differentiation.^53^ DART was also recently used to evaluate the effectiveness of different common laboratory gloves in preventing contamination of materials by permeating human sweat.^54^ The study evaluated the permeation of human sweat and chemical standards through nitrile, polyethylene and latex gloves, demonstrating polyethylene gloves exhibited the highest degree of permeation whereas double‐layered nitrile gloves offered the best protection. The study highlighted the importance of glove choice in both preventing contamination of forensic evidence but also protecting the wearer from hazardous materials encountered at crime scenes and in the laboratory.

### 3.2 Disease diagnostics

The timely detection of disease and infection is crucial in clinical diagnostics, ensuring medical problems can be identified and treated swiftly to achieve the best outcome for patients. The transportation and analysis of patient samples create a major bottleneck in current diagnostic workflows. As such, the development of techniques to supplement and even replace laborious laboratory analyses has led to a vast amount of research in the use of AIMS for disease diagnostics.

Given the significant burden of cancer on the worldwide population, the development of disease diagnostics is understandably dominated by cancer detection research, with a variety of AIMS techniques being applied to the detection of potential biomarkers for a range of cancer types. The development of a technique capable of detecting early‐stage cancer is a holy grail in cancer diagnostics, and ambient ionization may hold the key to making this a reality. PESI‐MS is a particularly popular choice for the analysis of potentially cancerous tissues, due to its simple operation and commercial availability. Wang et al. used PESI‐MS and machine learning to develop a method of detecting papillary thyroid carcinoma, demonstrating the ability to readily differentiate cancerous and benign thyroid nodules.^55^ In this study, solvent extractions of biological material were used, but PESI‐MS has been widely used for the direct analysis of tissue samples in the past. Hakoda et al. also used PESI with machine learning, this time targeting liver tumours.^56^ Through the analysis of almost 300 cancerous and healthy tissue samples, the authors demonstrated the technique to have a high sensitivity and specificity, with a diagnostic accuracy of 92%. PESI‐MS was also recently applied to the analysis of pulmonary tumors^57^ and liver tumours across different populations, highlighting its versatility with different tissue types.^58^ Although PESI has been widely applied to the analysis of cancerous tissues, it does still require tissue biopsy, as opposed to techniques such as REIMS which can be used in situ in the operating theatre and could feasibly be used for non‐invasive analysis.

**FIGURE 2:**
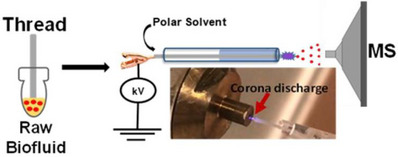
Schematic of cellulose thread microsampling and reactive thread spray mass spectrometry setup. Reprinted with permission from Swiner et al.^64^ Copyright 2022 American Chemical Society.

Katz et al. used picosecond infrared laser MS (PIRL‐MS) with a handheld sampling probe in the analysis of 97 *ex vivo* skin samples, including healthy skin, melanoma and squamous cell carcinoma.^59^ Constructing a principal component analysis‐linear discriminant analysis model, the three sample types could be readily distinguished within 10 s showing sensitivity and specificity as high as 95% and 98%, respectively. This protocol was capable of identifying a number of potential biomarkers for skin cancer. The authors furthermore evaluated the potential of using PIRL‐MS for in situ use through direct analysis of tumour, muscle and skin tissue of a murine model. The ability to use such a technique for the rapid analysis of skin in clinics would be a game‐changer for skin cancer diagnostics, enabling the screening of suspect growths prior to biopsy. SESI‐MS is particularly suited to the analysis of volatile compounds (VOCs) and was recently used to study the VOC profiles of different lung cancer cells (non‐small cell and small cell).^60^ The authors sampled headspace from lung cancer tissue cultures, detecting 60 VOCs associated with the cancer cells and demonstrating variation in the VOCs produced by the two types of the cancer cell. They furthermore showed that the VOCs produced by cancer cells were altered after exposure to cisplatin, a common chemotherapy treatment. Although this proof‐of‐concept study sampled from cell cultures, the compounds detected could be beneficial biomarkers in the development of breath‐based cancer diagnostics. Numerous other AIMS techniques have been utilized in the study of cancerous cells and tissues, including CBS for the study of brain tumours,^61^ PS‐MS to analyze urine samples from prostate cancer patients,^62^ and internal extractive ESI (iEESI) for colorectal cancer tissue analysis.^63^ Although cancer research accounts for the majority of AIMS‐based diagnostics development, there has been a focus on other diseases and conditions. Swiner et al. used reactive thread spray MS to study the serum metabolome of obese patients (Figure 2),^64^ Sarkar et al. used PS‐MS coupled with ion mobility for the diagnosis of Parkinson's disease using sebum,^65^ and Harkin et al. used LESA to image murine diabetic kidney tissue.^66^

In recent years, there has been increased interest in moving MS‐based clinical diagnostics from the laboratory into the operating theatre, particularly for the real‐time characterization of healthy and malignant tissue during surgery. By rapidly establishing whether tissue is healthy or cancerous, surgeons have an additional tool to ensure damaged tissue has been sufficiently excised and healthy tissue is not being unnecessarily removed. REIMS is undoubtedly the most frequently used technique for this application. In recent months, REIMS has been applied to the analysis of tissue during glossectomy surgeries^67^ and basal cell carcinoma resections^68^ in the operating theatre, whereas a method for analyzing brain tumours was also developed outside of the operating theatre through the use of tissue biopsies.^69^ Furthermore, REIMS has also been applied to the analysis of canine tissue samples for potential use in veterinary surgery.^70^ In all of these studies, REIMS and the classification models developed enabled the characterization of malignant tissue with high accuracy. Similarly, Yau et al. validated a REIMS method for the analysis of skin, with the intention of developing a method that could be used during tissue excision in burn cases.^71^ They compared REIMS lipidomics results with those obtained using LC‐MS/MS to confirm the agreement between the two techniques and demonstrated that both techniques could detect chemical differences between healthy and excised skin.

Finally, as novel clinical diagnostics are developed, there is a drive to create diagnostic methods that are rapid and non‐invasive, reducing the impact on patients. Exhaled breath is amongst the most attractive samples for this purpose, simply requiring the patient to exhale into a breath collection device or even directly into the analytical instrument. Numerous AIMS techniques have been applied to breath analysis, though SESI‐MS is by far the most common, with several applications in recent months. Wüthrich et al. applied the technique to study the effects of nutritional interventions on exhaled breath, performing real‐time analyses of participants’ breath before and after consuming a high‐energy shake.^72^ Numerous features were found to change in response to the intervention, including fatty acids, amino acids and metabolites linked to gut microbiome activity, demonstrating the potential to use AIMS for nutritional studies and monitoring of medical conditions necessitating dietary interventions. Other groups have applied SESI‐MS to identify exhaled breath biomarkers relating to conditions such as sleep apnea^73^ and cystic fibrosis,^74^ further demonstrating the versatility of the technique to study breath biomarkers of different diseases and disorders. Although less commonly utilized for breath analysis, other AIMS techniques are applied in this area including DART‐MS to monitor changes in breath metabolites throughout the day^75^ and LTP‐MS for the offline analysis of exhaled breath metabolites collected onto filter paper.^76^

### 3.3 Food and agriculture

Quality control of food and agricultural materials is critical in the detection of contamination and adulteration to prevent the distribution of hazardous or otherwise compromised food products. Analytical testing plays a vital role in this process, with gas chromatography‐MS and LC‐MS being crucial in identifying contaminants in food products, studying the flavour profile to ensure the taste and consistency of products, and validating the authenticity of a product to detect food fraud, to name a few. Given the high demand in this area of analysis, the ability to speed up workflows and perform on‐site testing has resulted in food and agriculture becoming one of the major fields for AIMS applications.

Perhaps the largest application of AIMS in food science is in the field of product authentication. Certain specialized food and drink products can fetch a high price, inevitably leading to the production of adulterated or counterfeit food products, often achieved using a lower‐quality substitute. The adulteration of animal products has been a particular focus of AIMS research. Although initially developed for the analysis of biological tissues during surgeries, REIMS has been increasingly utilized in this field of study, likely due to its user‐friendly interface and ease of use by non‐experts. Cardoso et al. used REIMS for the rapid analysis of beer, successfully differentiating between different products and demonstrating the potential to use AIMS techniques for quality control, flavour profiling and authentication of alcohol products.^77^ Zhang et al. used REIMS for the analysis of lamb products, demonstrating the detectable variation in products based on the sex of the animal, in addition to castration status, diet, and breed.^78^ REIMS has also been used for the chemical prediction of beef palatability, studying the chemical variation associated with factors such as flavour, juiciness, and tenderness, all of which will be important in the authentication.^79^ With similar efforts to improve the authenticity and quality of animal products, REIMS has similarly been used for the authentication of shrimp^80^ and beef,^81^ for the characterization of aged tuna products^82^ and fish speciation,^83^ and for milk authentication.^84^ Numerous other AIMS techniques have been demonstrated as potential tools in the authentication of food products, including ASAP for the authentication of Manuka honey^85^ and Chinese teas,^86^ DART for the discrimination of milk products^87^ and edible insects,^88^ DESI for the characterization of plant and animal‐derived milk,^89^ and desorption APCI for vanilla characterization.^90^ Bertella et al. performed a comparison of multiple AIMS techniques for wine authentication, evaluating PS, DBD, and LTP‐MS whilst comparing the results to a standard ESI‐MS method.^91^ Each technique had its own advantages, with plasma‐based techniques allowing for solvent‐free analysis but PS‐MS producing richer mass spectra with superior signal intensity.

Quality control is a critical step in the production and distribution of food products, particularly for identifying any potential contaminants or adulterants. The ability of AIMS techniques to achieve this in a rapid manner has obvious implications for the food production pipeline. In some regions, there has been an increase in the adulteration of herbal remedies and nutritional supplements, particularly in the illicit addition of pharmaceuticals such as weight loss drugs and blood pressure medication. In a recent study, ASAP was used for the rapid characterization of 42 illegal additives known to be added to plant‐based products, furthermore comparing the results against a standard LC‐MS/MS method.^92^ The study demonstrated the potential of ASAP both with analytical standards and the drugs spiked into a coffee sample to simulate the detection of the target analytes within a complex matrix. All but one of the analytes were detected in the coffee, albeit with notably higher LODs compared to standard solutions. Another study by Lin et al. focused on the detection of aldehydes in food, which can accumulate during product storage and have potentially harmful effects on consumers.^93^ They used reactive PS‐MS for the analysis of aldehydes in fifteen food samples, including fruit, meat and tofu products, demonstrating quantitation of four target compounds with good accuracy compared with an LC‐based method. Bisphenols are a group of molecules commonly found in plastics, dyes and resins and have known adverse effects on the human body, implicated in reproductive disorders, cardiovascular disease, and cancer. The potential for bisphenols to leech from food packaging into consumer products has thus been a matter of concern for several years, necessitating the development of rapid and robust techniques to detect their presence in food. In a recent study, PS‐MS was utilized in the detection of bisphenol A and bisphenol S in both UHT milk and its packaging.^94^ Milk was analyzed by application of the samples to the PS matrix whereas the packaging itself was cut into triangles and analyzed directly in lieu of the usual PS substrate. The bisphenols were readily detected in both the packaging and milk products, present as high as 151 ng/ml in milk samples. Other techniques have furthermore been utilized in the rapid detection of food and drink contaminants, including DART for animal feed analysis,^95^ EESI for arsenic detection,^96^ and CBS for the detection of food‐related mycotoxins.^97^

AIMS has proven to be particularly popular in the analysis of fruit and vegetable products. Birse et al. used DART‐MS coupled with a compact low‐cost mass spectrometer for the differentiation of organic and non‐organic leeks.^98^ The organic vegetables could be readily identified using an orthogonal PLS‐DA model with 94%–100% accuracy, demonstrating the potential for rapid authentication of organic products with semi‐portable instrumentation for on‐site analysis. The technique was furthermore applied to the analysis of agricultural products to characterize polyphenols, a group of compounds believed to be important in disease prevention.^99^, ^100^ iEESI‐MS has been used for the analysis of *Panax notoginseng*, a valuable herb used in traditional Chinese medicine whose quality and efficacy depend greatly on its growing conditions and thus its value.^101^ Using iEESI‐MS, the authors identified a number of sugars, organic acids, and saponins differentiating between different types of the herb, indicating the potential for rapid analysis techniques to replace the traditional laborious methods used to authenticate the quality of valuable herbs. EESI has been used for the detection of the disease Huanglongbing in citrus fruits.^102^ The authors were able to rapidly differentiate between healthy and diseased oranges, demonstrating the potential to use AIMS for rapid health monitoring of agricultural products. Other studies have used PS‐MS to study the effects of harvest time on the chemical composition of plant products,^103^ DESI to image the distribution of target compounds in developing soybean seeds^104^ and metabolites in traditional herbal medicine plants,^105^, ^106^ ASAP to chemically characterize different orange varieties,^107^ CBS to study pesticides on fruit,^108^ and air flow‐assisted ionization MS imaging to study the distribution of metabolites in different regions of the mango fruit.^109^

### 3.4 Pharmaceuticals

Ambient ionization has also found utility in the analysis of pharmaceutical products, enabling the rapid characterization of drug products. In addition to the direct analysis of tablets and liquid medicines, AIMS has further utility in the identification and quantification of pharmaceuticals in biological fluids, such as blood, for therapeutic drug monitoring. This involves the real‐time measurement of drugs in the body in order to ensure appropriate concentrations of prescribed medicines are present in a patient's body to inform on dosage schedule and adherence, in addition to screening for instances of a drug overdose.

**FIGURE 3:**
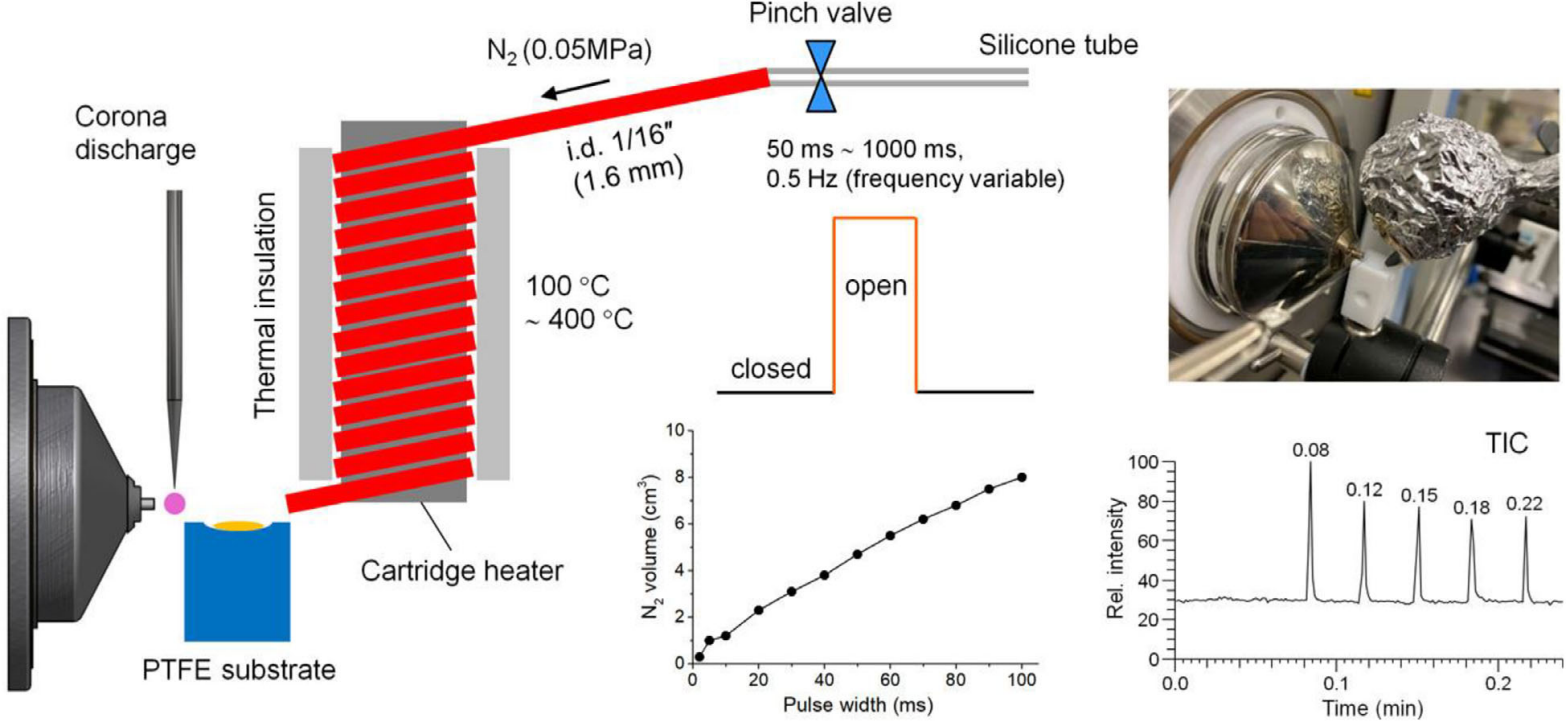
Schematic of the heat pulse desorption system for the desorption and ionization of low‐volatility compounds. Reprinted with permission from Ninomiya et al.^110^ Copyright 2022 American Chemical Society.

For the rapid characterization of pharmaceuticals, Ninomiya et al. developed heat pulse desorption MS.^110^ In this technique, the liquid or solid sample is applied to a surface and a very brief (50 ms) pulse of heated N_2_ gas (350°C) is used to rapidly desorb analytes (Figure 3). Analytes are then immediately ionized by corona discharge and drawn directly into the mass spectrometer for analysis. In a recent study, numerous pharmaceutical tablets were directly analyzed (carbocysteine, etizolam, sildenafil, and erythromycin) in addition to a commercially available liquid anti‐fungal treatment, all of which were readily detected. The technique was also demonstrated to be suitable for the analysis of explosives and illicit substances. ASAP has also been utilized for the rapid characterization of medicinal products. A recent study demonstrated its utility in quantifying the amount of melatonin, a hormone commonly used to facilitate sleep, in over‐the‐counter medicines, comparing the results with a standard LC‐MS method with equivalent results.^111^ The technique was similarly used to measure sildenafil, more commonly known as Viagra, in herbal medicines promising to treat erectile dysfunction, demonstrating the variation in the presence and abundance of the active ingredient in 12 different medicines purchased online.^112^ Other AIMS techniques developed for direct pharmaceutical analysis include the coupling of a DBD ion source with ion mobility MS for the rapid analysis of several painkillers, allergy treatments and antibacterials,^113^ and the use of DESI‐MS high‐throughput screening for drug discovery.^114^

The analysis of pharmaceuticals in biological materials has gained more attention. Shamreva et al. developed a sampling probe for the collection, storage, and subsequent ambient ionization of body fluids containing drugs, with the specific aim of developing lightweight and compact sampling probes that could be used to collect and store specimens from astronauts during spaceflight.^115^ Blood and saliva samples spiked with acetaminophen as a model drug were placed in the polypropylene sample probes and stored for different periods of time. The probe itself then becomes the ionization source, with the application of solvent and a high voltage to the probe resulting in the formation of an electrospray. The drug could be detected at concentrations below the lower therapeutic limit in human biofluids with good linear response, indicating its suitability for semi‐quantification. In terms of sample storage, the technique offers similar advantages to the use of dried blood spots, though in this study samples were only evaluated after storage for several days, far short of the several months that samples would likely be stored for monitoring during spaceflight. In a recent study by Rocca et al., a DESI‐MS method was developed for the detection and quantification of 11 ototoxic and narcoleptic drugs in dried blood spots.^116^ Using an automated system to locate and ionize lines of multiple samples and replicates, the technique demonstrated the ability to rapidly target and detect multiple classes of drugs with high precision and accuracy in comparison to a standard LC‐MS/MS method. PS‐MS has also been used for the detection of pharmaceuticals in biological fluids, being used for the detection of remdesivir and its active metabolite in plasma^117^ and coupled with a miniature MS for the detection of HIV drugs in whole blood.^118^

The physical distribution of drugs in the body can also provide critical information and various MS imaging techniques have been developed for this purpose, typically utilizing DESI‐MS and animal models. Sanchez et al. used nano‐DESI to image the distribution of diclofenac, an anti‐inflammatory drug, in the kidney and liver of mice.^119^ Despite challenges with low signal intensity and severe ion suppression, the authors were able to demonstrate the localization of drug metabolites in specific regions of the kidney and widespread distribution throughout liver tissue. DESI and matrix‐assisted laser desorption ionization (MALDI) was also recently used to study the distribution of therapeutic peptides in a murine model.^120^ The study imaged the immunosuppressant cyclosporine and its metabolites throughout the internal organs, demonstrating the drug itself was primarily detected in the pancreas and liver, whereas metabolites were primarily found in the intestines. Several other studies have used DESI‐MS imaging to study the distribution of drugs in tissues, including acetaminophen and its metabolites in the mouse liver^121^ and extracellular signal‐regulated kinase inhibitors in mouse brain tissues.^122^ Finally, another recent study utilized SESI‐MS for the real‐time detection of inhaled aerosols in exhaled breath for the purpose of studying the aerosolization performance of inhaled drug products.^123^

The detection of pharmaceuticals in animals also has implications for environmental challenges. The presence of drug products in marine life can provide critical information regarding the contamination of water supplies and the effects of contaminating drugs in the body. Zhao et al. used DESI imaging to visualize the distribution of the anti‐anxiety drug Diazepam in Zebrafish through exposure to water, following reports of the contamination of water supplies with the drug and its byproducts.^124^ In visualizing the distribution of the drug and metabolites in the body, the authors demonstrate the potential to use MS imaging to study how marine life can be affected by water contaminants and how these products could behave in the human body if the exposure occurred, either through consumption of the contaminated water or affected animals.

### 3.5 Biological materials

Due to the complexity of biological matrices, such as blood and tissue samples, traditional MS techniques typically require extensive analyte extraction and sample cleanup prior to analysis, rendering the analysis of biological materials time‐consuming and costly. The advent of AIMS has provided a means of enabling the direct analysis of biofluids, tissues, and even whole animals, providing high‐throughput, real‐time testing.

Methods for preparation‐free biofluid analysis have great utility in clinical diagnostics and forensic science. As such, numerous AIMS techniques have been evaluated for the direct analysis of raw or minimally‐prepared biological liquids. Chen et al. developed a technique called thread‐based isotachophoresis DESI‐MS (TB‐ITP‐DESI‐MS) for the analysis of alkaloids in biological fluids.^125^ The technique used a fabric thread to act as an electrofluidic device for on‐thread separation, after which a DESI source scans along the thread for analyte desorption and ionization. In the analysis of raw urine spiked with a number of alkaloids, sensitivity was 5–11 times greater compared with standard DESI. Yang et al. developed a modified form of PS‐MS for the detection of miRNA in whole blood, incorporating an on‐paper DNA‐strand‐displacement reaction enabling the quantification of miRNAs in the sample.^126^ Finally, a PS‐MS method utilizing surface‐modified hydrophobic paper to enable the collection of 3D spheroid blood droplets was developed with the intention of avoiding the volcano effect experienced when blood is collected onto DBS cards.^127^ This was applied to the analysis of creatinine in whole blood, exhibiting comparable results to a standard LC‐MS/MS method.

The ability to desorb and ionize analytes in situ has naturally led to AIMS being a popular tool for the characterization of large molecules in complex biological matrices, thus a large number of studies have harnessed these techniques for peptide and protein analysis. Hale et al. recently applied both nano‐DESI and LESA for the analysis of protein assemblies in liver, kidney and brain tissues.^128^ They demonstrated the ability to detect and identify intact endogenous protein assemblies across the range of 37 to 145 kDa in size. The same group have further utilized this combination of technologies to study protein‐ligand interactions,^129^ to image ligand‐ and metal‐bound proteins in rat brains^130^ and to study drug‐protein complexes in the liver of rats.^131^ Another group developed a nano‐DESI imaging with an on‐tissue top‐down proteomics method for the proteoform‐selective imaging of rat brains, visualizing proteoform distribution across tissues.^132^ Finally, both LESA‐ion mobility spectrometry (IMS)‐MS and MALDI imaging have been evaluated for the analysis of hormones in tissue.^133^ Formalin‐fixed, paraffin‐embedded (FFPE) tissues are typically used by hospitals for histological analysis, however, this format of samples has traditionally been avoided in mass spectral imaging studies in favour of fresh or fresh frozen tissues. This study specifically focused on the analysis of FFPE tissues, developing workflows that could be applied to this material and demonstrating the utility of implementing multiple complementary techniques.

The ability to perform rapid tissue imaging has become one of the major applications in AIMS research, with DESI‐MS being the most popular technique for this purpose. The past 12 months have seen an abundance of studies harnessing DESI for imaging of biological tissues, particularly in murine models, such as imaging of alkaloids in rat brains,^134^ visualizing the distribution of carcinogens in the kidneys of rats,^135^ and imaging of mouse and rat brains with convolutional neural network‐based deep learning.^136^ In addition, numerous modifications to the original DESI technique have emerged to improve mass spectral imaging capabilities. In a recent study, the use of desorption electro‐flow focusing ionization, a DESI variant, was demonstrated with biological tissues, incorporating an electro‐flow focusing nebulizer to achieve improved focusing of the spray solvent.^137^ Wu et al. used imprint DESI with post‐photoionization (DESI/PI) to study plant metabolites, a technique in which, rather than imaging from the sample directly, the tissue metabolites are transferred to an imprinted substrate prior to analysis, maintaining the chemical composition and spatial distribution of the original sample.^138^ In doing this, the authors were able to image a broad range of analytes, including terpenoids, amino acids, lipids, tannins and alkylphenols. The same group has also recently applied DESI/PI to image various endogenous metabolites and dopants in the brains of mice.^139^, ^140^ An increasing number of workflows are also being automated to reduce manpower hours and increase throughput. In a recent study, an automated DESI system was developed for the rapid analysis of tissue microarrays, which are often used for the large‐scale analysis of biological tissue samples.^141^ In this study, the system was able to process a tissue microarray of over 6000 samples with a highly impressive analysis time of less than 1 s per sample. The more familiar DESI variant nano‐DESI is also frequently used for the imaging of biological materials. Mavroudakis et al. used nano‐DESI to study alkali metal ions by supplementing the solvent with crown ether molecules, which form complexes with alkali metal ions.^142^ In doing so, they demonstrated the first use of crown ethers in MS imaging to enable the simultaneous detection of elemental and metabolite species. Hu et al. utilized a recently developed deep learning approach for dynamic sparse sampling with nano‐DESI for the imaging of mouse kidney tissue.^143^ The technique reduces the number of mass spectral measurements required, thus improving the throughput of imaging experiments by at least 2‐fold. Li et al. used nano‐DESI with an integrated microfluidic probe designed to improve the stability of the liquid bridge used for analyte extraction,^144^ demonstrating the effectiveness of this device through imaging mouse tissue sections.

AIMS has furthermore found utility in the study of plants and non‐human animals. Insects are known to secrete a complex blend of pheromones critical for communication, the analysis of which can be incredibly challenging due to their transient nature. DART‐MS in particular has proven to be a popular tool for the characterization of insect pheromones and metabolites. In a recent study, the technique was used for the analysis of pheromone glands in *Drosophila melanogaster*, more commonly known as the fruit fly.^145^ Highly rich species‐specific lipid profiles could be measured, consisting of wax esters, fatty acids, diacylglycerides, and triacylglycerides. Morgan et al. applied REIMS to the direct analysis of mosquito larvae to study insecticide resistance, demonstrating distinct chemical variation between insecticide‐resistant and insecticide‐susceptible strains.^146^ Furthermore, the ability of AIMS to achieve non‐destructive, in situ, analysis opens up the fascinating possibility of performing real‐time analysis on living animals. Kriger et al. used the MasSpec Pen, a device traditionally utilized for clinical diagnostics, for the analysis of poison frogs.^147^ Poison frogs harness the ability to excrete different alkaloids as a chemical defence against predators. By applying the MasSpec Pen directly to the skin of the live frog, the authors achieved the measurement of target alkaloids throughout a month‐long feeding study, in addition to performing untargeted analysis of skin excretions. Other techniques have also been demonstrated in the analysis of plant metabolites, with Walton et al. using the liquid micro junction‐surface sampling probe (LMJ‐SSP) to study the spatial distribution of amino acids in plant material and bacterial biofilms^148^ and Nakata et al. using picolitre pressure‐probe electrospray‐ionization MS to study the effects of salt stress in single plant cells.^149^

The early identification of bacterial species is of crucial importance for rapid diagnostics in order to ensure appropriate and timely treatment of infections. As such, AIMS can provide the means to characterize microbial species in near‐real time. Bacteria and other microbes produce a variety of volatile metabolites which may be species‐specific and thus could provide a means of rapid identification from cell cultures. SESI‐MS was recently utilized for the rapid characterization of VOCs from *S. aureus* and *S. pneumoniae* cultures, demonstrating the ability to differentiate between the two within minutes.^150^ Povilataitis et al. achieved similarly impressive results with the MasSpec Pen for the characterization of *S. aureus*, *Streptococcus*, *and K. kingae*.^151^ PS‐MS has been used for the species determination of microbes, as a standalone technique to identify metabolites in fungi^152^ and by coupling the AIMS technique with ion mobility to achieve separation of phospholipid isomers to provide improved differentiation of species.^153^ In this PS‐IMS‐MS study, the technique was able to differentiate five different *Bactillus* species after only 4 h of cell incubation and a 2 min analysis time. Characterization of phospholipids in bacterial cultures has also been achieved with DESI‐MS, achieving an impressive sample analysis time of less than 10 s.^154^ The analysis of bacterial species has primarily targeted smaller metabolites produced by microbes, though a recent study used LESA‐MS for the detection of proteins in *E. coli* colonies.^155^ Through the combination of electroporation to release proteins from cells followed by LESA‐MS, intact protein assemblies up to 50 kDa could be detected directly from growing colonies. The analysis of biological materials is perhaps the largest area of research utilizing AIMS technology, and this section is by no means exhaustive. Nevertheless, additional biological applications worthy of note include the development of a high‐throughput DESI method for the exploration of enzymes as potential drug targets,^156^ and the use of DART and ASAP to characterize the metabolome of lichens.^157^

### 3.6 Miscellaneous applications

The majority of studies utilizing ambient ionization can be broadly categorized into the aforementioned fields of study. Nevertheless, the versatility of AIMS is increasingly demonstrated by the more niche areas of research in which these techniques are being utilized. The field of polymer chemistry has not seen many applications of AIMS, however, in recent months, the potential power of rapid analysis is becoming apparent. A recent study coupled ASAP with IMS and tandem MS to study thermoplastic elastomers, a type of copolymer with various industrial uses.^158^ Given the complexity of these materials and the high molecular weight of the polymers, traditional analysis has been challenging and time‐consuming. In this study, the authors demonstrated that ASAP provided a simple means of studying the thermal degradation of the elastomers, enabling the study of degradation products as the samples were exposed to increasing temperatures. Furthermore, laser‐assisted micro‐pyrolysis with FAPA‐MS has been used for the 3D imaging of polymers and polymer additives, enabling the thermal separation of components prior to analysis.^159^ DART and DESI have also been utilized in polymer analysis, for the study of the miscibility of polymer blends^160^ and the analysis of sequence‐defined polymers, respectively.^161^ In recent years, ambient ionization has also been recognized as a powerful tool for the rapid and real‐time study of chemical reactions. A class of compounds called phthalimides has huge potential in pharmaceuticals given their broad range of applications, however, their synthesis and analysis are traditionally extremely time‐consuming. In a recent study utilizing reactive PS ionization, the authors were able to quickly explore different experimental parameters and reaction products by performing in situ reactions on the PS substrate.^162^

The use of AIMS in environmental sciences has also exhibited some slow but steady interest, with numerous techniques being evaluated for their utility. Sedimentary rocks are known to contain significant amounts of oil held within the many pores of the rock; however, the extraction of this oil is extremely challenging and inefficient. As such, there has recently been interest in understanding the surface chemistry of these materials to facilitate the development of improved extraction methods. A recent study leveraged both DESI‐MS and LAESI‐MS to spatially map both polar and non‐polar metabolites on the surface of two different types of mineral rock.^163^ DESI revealed the distribution of fatty acids and a disaccharide across the rock surface, whereas LAESI revealed the presence of hydrocarbons. Although a somewhat niche area of study, the research demonstrates the versatility of AIMS for environmental applications and the power of combining techniques to target a broader range of analytes. Other studies have further evaluated AIMS for different applications to environmental samples, including the use of DART to detect anatoxin‐a, a neurotoxin produced by cyanobacteria, in field samples,^164^ the use of iEESI to detect perfluorooctanoic acid contamination in fish,^165^ and the use of a dual plasma ion source (incorporating both corona discharge and a microwave plasma torch) to detect trace sterols in urban water samples.^166^

Plasma‐based AIMS techniques have also been utilized for the analysis of complex oil products. Fully formulated oil (FFO) is a substance frequently used in the automotive industry, but its high chemical complexity makes full characterization challenging and traditional techniques typically target specific analytes. In a recent study, DBD‐MS was developed for the rapid untargeted analysis of FFO, demonstrating the ability to detect a broad range of components within the oil in <1 min.^167^ Finally, a recent study has seen DART‐MS utilized for the rapid quality control of cigars. Given the potentially high value of certain cigar brands, the product is inevitably at risk of fraud. The recent study used DART‐MS for the discrimination of different tobacco leaf products, to study the effects of different leaf pretreatments, to determine nicotine content, and to ascertain the geographic origin of the product, all of which could be achieved. The analysis was also compared with an LC‐MS method, but only DART could determine the geographic origin and pretreatments.^168^

### 3.7 Technological and method developments

Despite the presence of AIMS for almost two decades, the field is still in its infancy and has a long way to go before achieving the same robust standards as traditional techniques. Still, each year brings a wealth of new research striving to drive the field forward. The introduction of entirely new AIMS techniques has perhaps slowed in recent years, as efforts are increasingly focused on the improvement and enhancement of the existing tried and tested technology, though some novel techniques and variants of existing techniques have nevertheless emerged.

**FIGURE 4:**
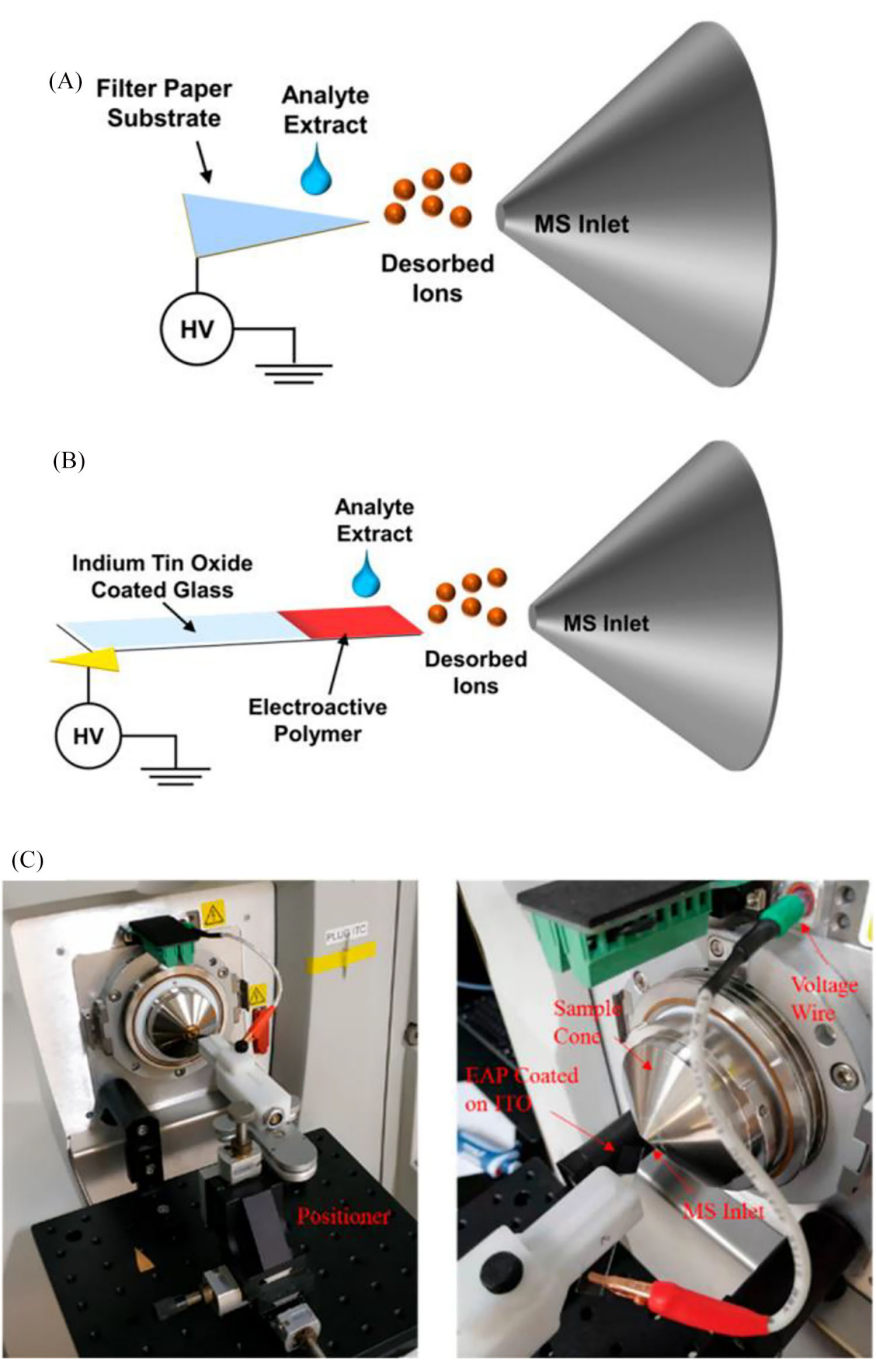
Schematic of paper spray ionization in comparison to electroactive polymer‐based spray ionization. Reprinted with permission from Wang et al.^169^ Copyright 2022 American Chemical Society.

Wang et al. introduced electroactive polymer‐based spray ionization (EAPSI), a technique aiming to improve the performance of PS ionization (Figure 4).^169^ In EAPSI, an indium tin oxide‐coated glass slide is used as an electrode onto which the electroactive polymer and sample are applied, before the application of a high voltage to induce electrospray as in PS‐MS. Compared to PS‐MS, EAPSI achieved improved LODs and limits of quantitation for solvent‐based analytes, and similar performance for analytes in complex matrices (artificial saliva and urine). Pursell et al. developed vibrating sharpedge spray ionization (cVSSI) coupled with APCI to specifically target analytes susceptible to ion suppression and matrix effects.^170^ The device consists of a glass emitter through which liquid samples are introduced and a piezoelectric transducer to induce the nebulization of samples. The technique can be used with or without the application of a high voltage depending on the target analytes. In a comparison of cVSSI with standard ESI, cVSSI appeared to reduce ion suppression effects and achieved an overall boost in the signal of all ions by approximately three orders of magnitude.

Li et al. developed desorption, separation and ionization MS (DSI‐MS), a tool which combines a heated sample holder for analyte desorption, a sand core filter to provide a porous surface area for differential analyte desorption, and an atmospheric pressure glow discharge source.^171^ In a recent study they analyzed urine samples spiked with 2‐dimethylaminoethylamine to achieve the derivatization of analytes with carbonyl, ketone and aldehyde groups and subsequent differential desorption and ionization of urine metabolites, enabling the characterization of healthy and bladder cancer urine samples. In order to reduce ion suppression effects caused by salts, heat‐assisted dual neutral spray ionization was also recently introduced.^172^ This technique incorporates a dual spray system and a heated plate onto which sample salts crystallize, enabling online desalination prior to analyte analysis.

Finally, some groups have endeavoured to create new sampling strategies that can feasibly be coupled with multiple types of ambient ion sources. R‐sniffing MS utilizes a Venturi pump for constant sampling, enabling either direct gas analysis or aspiration of a solvent droplet applied to a surface for the analysis of solids.^173^ In this study, the device was coupled with ESI and APCI, but could feasibly be integrated with other AIMS techniques. Modifications of the ASAP probe have also been developed for improved sample delivery. Strong et al. developed a cost‐effective probe for the introduction of air‐ and moisture‐sensitive compounds,^174^ whereas a separate study developed the atmospheric breath analysis probe to enable the direct introduction of exhaled breath into the ion source.^175^

Other studies have focused on the improvement of existing techniques. Given the popularity of PS ionization, numerous studies have focused on enhancing the performance of this technique and resolving some of its deficiencies, for instance by evaluating the effects of different solvents, paper types and spray modes.^176^ Brown et al. conducted a thorough evaluation of optimal parameters for cone spray ionization, a recently developed PS variant.^177^ The study involved the evaluation of various solvents, conductive plastic materials, and cone geometries, in addition to the development of a custom‐built autosampler to improve sample throughput, solvent delivery, and the reproducibility of cone positioning. Frey et al. has focused on improving PS ionization for the analysis of dried blood spots.^178^ In a recent study, they introduced pinhole PS ionization, a new modification of the technique developed to facilitate the analysis of dried blood spots. The technique involves the use of embossed hydrophobic paper strips onto which patient blood samples are applied and then stored, transported or immediately analyzed. For analysis, the dried blood spot is punched, inverted 180° and placed on a second piece of triangular hydrophobic paper. A hypodermic needle punctures the bottom of the well holding the DBS, creating a pinhole channel to guide the spray solvent through the blood spot. The application of solvent and high voltage then results in electrospray as per traditional PS‐MS, achieving sample analysis in less than 1 min, though with significantly lower sensitivity than the LC‐MS/MS method used for comparison. Finally, Foest^179^ and Seró^180^ et al. each respectively coupled PS‐MS with a flexible microtube plasma and atmospheric pressure photoionization to facilitate the ionization of less polar analytes, Wang et al. used carbon fibre as the PS substrate to enhance sensitivity and signal stability,^181^ whereas Martínez‐Jarquín et al. introduced an aptamer‐functionalized paper substrate for the concentration and detection of small molecules.^182^

Similar efforts have been made to improve the performance of other commonly used techniques. CBS ionization has been improved via studies examining the effects of spatial positioning on performance,^183^ modifications to the CBS blade design to improve reproducibility,^184^ and the development of blades coated with a monoclonal antibody layer to produce an immunoaffinity blade spray technique.^97^ Lin et al. performed an evaluation of the effects of different solvent systems and extraction times on the extraction and detection of different molecules.^185^ Chemical profiles were notably affected by the use of different solvent systems and extraction times, highlighting the importance of extensive optimization when developing solvent‐based AIMS techniques. Krenkel et al. developed an ultrahigh‐throughput liquid atmospheric pressure MALDI approach for the analysis of peptides and enzymatic assays, achieving the impressive analysis of up to 60 samples per second under some conditions.^27^ Other studies aiming to improve existing techniques have introduced a plasma treatment step to the DESI pipeline to increase ion signal,^186^ studied the effects of ion source geometry on the reproducibility of LAESI imaging,^187^ incorporated the use of helium as a nebulizing gas into DESI and ESI,^188^ and developed a post‐acquisition mass recalibration method to reduce mass drift in DESI analysis.^189^ Others have built custom REIMS devices using a soldering iron to improve analysis of poorly conductive samples,^190^ incorporated an online derivatization step into multiphase flow EESI for the analysis of reactive sulfur species,^191^ and modified the DART source to reduce undesirable oxidation when using nitrogen gas in place of helium.^192^ Some researchers have attempted to produce simpler and lower cost versions of AIMS techniques, such as using repurposed americium‐241 from smoke detectors as ion sources^193^ and building miniaturized sample nebulizing devices using cheap piezoelectric materials.^194^

Finally, other studies have coupled existing AIMS techniques with supplementary technologies. A handheld LMJ‐SSP was coupled to a miniature mass spectrometer via 50 cm flexible tubing to enable in situ surface analysis in the field.^195^ The technique was evaluated for the analysis of illicit drugs on skin and pharmaceuticals in biological fluids achieving an analysis time of <2 min and LODs as low as 5 pg. Ismaili et al. coupled LTP‐MS with IMS for the analysis of liquid pharmaceuticals and illicit drugs.^196^ In this setup, vaporized samples are exposed to the LTP ion source before immediately entering the drift tube of the IMS, with a reduced plasma gas to prevent interactions between the plasma and the IMS. In comparison to other techniques, such as LTP‐MS and ESI‐IMS, similar LODs could be achieved for a number of target analytes, including codeine, papaverine, and caffeine. In a similar vein, PS and leaf spray ionization techniques were also coupled with ion mobility and MS.^197^ In coupling these techniques, the authors demonstrated the ability to separate isomeric pesticides and lipids, reduce the effects of matrix effects, and improve the signal‐to‐noise of target analytes.

## 4 SUMMARY AND OUTLOOK

The introduction of ambient ionization has transformed the way analytical chemists solve problems. No longer are analysts restricted by the costly and time‐consuming processes traditionally used, and the potential to take MS into the field holds exciting promise for the future. This review has merely scratched the surface of the achievements in ambient ionization over the past 12 months, briefly touching upon the novel techniques and many exciting new developments introduced by analytical scientists. Recent advancements have particularly focused on the improvements of existing techniques, either through minor modifications of ion sources or the novel coupling of different techniques. Furthermore, some studies have notably pushed the limits of how many samples can be analyzed in a short space of time, with one study achieving the analysis of up to 60 samples in a second.^27^

The increasing enthusiasm surrounding AIMS is evident throughout the scientific community. MS societies in particular have demonstrated their interest in and support of AIMS in recent years, with the American Society for MS forming the Ambient Sampling and Ionization interest group and the British MS Society holding regular one‐day AIMS meetings as part of its Ambient Ionization Special Interest Group. In addition, both organizations have hosted ambient ionization sessions at their annual conferences. Instrument manufacturers have clearly recognized the need for ambient MS, with an increasing number of AIMS techniques being commercialized, the most recent addition being Waters RADIAN ASAP‐MS in 2021.

The recent novel applications and developments in AIMS indicate the future directions of the field and allude to the areas that could benefit from these techniques. The recent use of ASAP to analyze anabolic steroid esters, commonly used as performance‐enhancing drugs, introduces the potential of ambient ionization in anti‐doping.^45^ The challenge of detecting controlled substances in athletes is a widespread problem, and yet ambient ionization has been surprisingly unexplored in this field, despite the large body of research demonstrating the utility of AIMS in bioanalysis in other fields. In terms of technological developments, an increasing number of studies have coupled AIMS with IMS.^65^, ^113^, ^153^, ^197^ Given the lack of analyte separation in ambient ionization techniques, the differentiation of isomeric species is a major challenge. An AIMS‐IMS combination has the power to mitigate one of the major weaknesses of AIMS whilst not severely impacting its potential as a portable analysis technique due to the simplicity and compact size of many IMS devices. Finally, the ability of AIMS to achieve non‐proximate sampling enables the possibility of analyzing whole organisms. AIMS has been demonstrated in the analysis of live bacterial colonies for several years, however, more recent studies have applied ambient ionization techniques to the direct analysis of much larger species, including mosquito larvae^146^ and even living frogs.^147^ The ability to perform in situ non‐destructive analysis offers the exciting potential to study the real‐time chemistry of living organisms and gain a fascinating insight into animal biology.

Despite the growing enthusiasm for ambient ionization, there is still extensive research needed before AIMS can be widely adopted. The coming years will undoubtedly see the expansion of direct analysis into further fields of research, the adoption of AIMS techniques by laboratories outside of exploratory research, and the overall expansion of this revolutionary branch of analytical chemistry.

## AUTHOR CONTRIBUTIONS

Stephanie Rankin‐Turner: Writing—original draft (lead), Conceptualization (equal).

Patrick Sears: Writing—review and editing (equal).

Liam M Heaney: Writing—review and editing (equal), Conceptualization (equal).

## CONFLICT OF INTEREST STATEMENT

The authors declare no conflict of interest.

## Data Availability

Data sharing is not applicable to this article as no new data were created.
